# Knowledge transfer as a tool towards improvement of cancer care in low- and middle-income countries. 6th European Roundtable Meeting (ERTM), June 14th, 2019, Berlin, Germany

**DOI:** 10.1007/s00432-020-03209-7

**Published:** 2020-04-08

**Authors:** O. Ortmann, J. Torode, W. Schmiegel, C. Thomssen, M. Hakama, P. Basu, U. Ringborg, U. Helbig, For the participants of the ERTM, Matti Hakama, Partha Basu, Marjetka Jelenc, Josep Borras, Ulrik Ringborg, Julie Torode, Jan-Willem Coebergh, Johannes Bruns, Markus Follmann, Ulrike Helbig, Andreas Hochhaus, Monika Klinkhammer-, Margarete Landenberger, Olaf Ortmann, Wolff Schmiegel, Christian Thomssen

**Affiliations:** 1 German Cancer Society, Berlin, Germany; 2 Department of Gynecology and Obstetrics, University Medical Center Regensburg, Landshuter Straße 65, 93053 Regensburg, Germany; 3 Union for International Cancer Control (UICC), Geneva, Switzerland; 4 University Clinic at the University Hospital Knappschaftskrankenhaus Bochum, Bochum, Germany; 5 University Clinic and Polyclinic for Gynaecology, Martin-Luther University Halle-Wittenberg, Halle (Saale), Germany; 6 The Finnish Cancer Registry, Helsinki, Finland; 7 International Agency for Research On Cancer, World Health Organisation, Lyon, France; 8 EUROCAN Platform, Cancer Center Karolinska, Stockholm, Sweden; 9 Deutsche Krebshilfe, Berlin, Germany

**Keywords:** Cancer care, Knowledge transfer, Low-income countries, Middle-income countries, Education programmes, Quality, National cancer control plan

## Abstract

**Purpose:**

To identify key factors for the best practice of knowledge transfer from high-income settings to low- and middle-income settings.

**Results:**

Interactive sessions led to the identification of European learnings that can and should be shared beyond Europe. Furthermore, methods were characterised which may lead to successful knowledge transfer with subsequent quality improvement.

**Conclusion:**

To ensure successful implementation of knowledge and new methods, political support is extremely important. A strong focus should be an improvement of collaboration and network development. Rehabilitation, early and late pallative care, cost effectiveness and long-term follow-up are priorities. Limitations are budget constraints which limit the execution of NCCPs.

## Introduction

The German Cancer Society (DKG) and the Union for International Cancer Control (UICC) initiated European Roundtable Meetings (ERTMs) with the goal of sharing ideas on applied strategies and best practice to identify key instruments for improving quality of cancer care. This series started in 2014. Participants from different European countries and institutions discussed health structures and transformation of the theoretical health care standards into practical approach. Further meetings described central procedures and communication networks in cancer centers including patient pathways, consideration of the patients´ perspectives, needs for quality control to improve cancer care and factors for successful integration of translational science into oncology care concepts (Ortmann et al. 2015, 2016, 2017, 2018, 2019).

On the 14th June 2019, participants from European organisations met in Berlin for the sixth in a series of European Roundtable Meeting, focussing and sharing best practice for improvement of cancer care.

The 2019 roundtable focused discussions around best practice for knowledge transfer from high-income settings to low- and middle-income settings, asking the question: What knowledge transfer experiences and methods have facilitated quality improvement in cancer care services?

Following a welcome from Professor Olaf Ortmann and Dr. Julie Torode on behalf of the German Cancer Society and UICC, keynote presentations framed the issue from three perspectives: formalised education programmes in Germany, bilateral collaborations with Ethiopia and India, and international initiatives to improve quality and coverage of services.

## The role of educational programmes to improve cancer care, Prof. Dr W. Schmiegel, Germany

Professor Schmiegel took the example of cancer across the European Union to underscore inequalities in the region: while low- and middle-income countries in Europe currently have approximately only half of the new diagnoses annually compared to higher income settings, they carry 2/3 of the mortality, suggesting that positive trends in cancer prevention and control mirror the health expenditure.

Therefore, the opportunity to use expertise across Europe to make sure that no country is left behind, makes sense. Regular education can share, for example, evidence-based guidelines; quality indicators, certification systems; cancer registration and epidemiology skills and collaborative research can address gaps and barriers. Schmiegel emphasised that guidelines and other expertise are not a solution in themselves, they need to be integrated into a system, with defined performance indicators and audits for transfer of this best practice into routine care. Bringing clinical services and public health systems together to move away from the mindset of more patients equals more expense to quality of care and incremental improvement of patient outcomes.

While the network of certified cancer centres is now the standard and driver of quality cancer care in Germany and other high-income settings, many countries lack trained personnel and the multidisciplinary team approach, which is the core of their success. We must, therefore, work alongside the first cancer centres, as they are getting started, to help them be the reference centre for national planning—framing the question for the workshop—how do we transfer understanding, adaptation and stepwise introduction of the elements of robust guidelines and monitoring and evaluation of their implementation to countries starting out on this pathway?

## Learning models for quality improvement: the example of Ethiopia, Prof. Dr. C. Thomssen, Germany

Professor Thomssen reported from a long-term collaboration between the University of Halle in Germany and partners in Ethiopia at the three University sites in Addis Ababa and Gonder. Building on relationships between the former East Germany and Ethiopia, the gynaecology team has been collaborating since 15 years, beginning with obstetrical projects of quality assurance and a local breast cancer registry in a peripheral hospital. The reanalysis of tumor specimen demonstrating a high proportion of estrogen receptor positive breast cancer cases lead to a Tamoxifen donation project. A portfolio of work supports the population-based cancer registry and research as well as the training of clinical skills, linking to German networks as well as international groups like International Gynaecologic Cancer Society is needed. A key learning for us is that we have to adapt our approach, based on the situation. For example, we found that 34% of women present at the hospital in Addis Ababa with stage IIIb cervical cancer, but due to the long waiting times for radiotherapy, this had shifted to 64% of women by the time they actually accessed treatment. We have, therefore, worked with the local team to identify cases where neoadjuvant therapy could potentially lead to an operable cancer and have established an effective protocol.

Thomssen described key factors for success of the collaboration as:

- Always working with local leadership, citing the work on standards and quality of clinical care though a collaboration with the University to establish a school of public health;
- The cross-team support and engagement for the partnership in Ethiopia from staff in Halle;
- Running research alongside training efforts to build capacity, but also to identify the real barriers to implementation of best practice;
- Finding interim solutions such as working with the European School of Obstetrics and Gynaecology (ESOG) for the certification of trainees prior to the establishment of national board exams for gynaecological oncology in Ethiopia;
- Training physicians and technicians in tandem, with robust linkage to pathology and laboratory services;
- Create a positive environment for self-help, enthusiasm for improving care and publication;

A key challenge is “brain drain” of trained experts to roles outside of the country or to the private sector. While resources are limited in Ethiopia, specialization on a high level of gynaecological oncology is feasible. We are now striving hard to develop structures that will sustain the successes and integrate these into routine care for future impact, says Thomssen.

## Transfer of knowledge by training and education: experience in India, Prof. M. Hakama, Finland

Similarly, Professor Hakama shared that the collaboration between the University of Tampere in Finland with partners in India is a long-term commitment which began in the early 1990s. Knowledge is the driver of cancer control, so our aim was establishing the knowledge base. Supported financially by the Cancer Society of Finland, the collaboration began with a focus on doctorate training of epidemiologists. Putting capacities in place for a cancer registry in a region of India, supported by research funding, proved to be the start of establishing broader cancer prevention and control infrastructure, explained Hakama.

Reviewing the cancer data, priorities were clear—tobacco control and establishing early detection and screening services for high-burden cancers such as cervical cancer, as well as palliative care. Back then, we had to challenge our own approach to tobacco control, as we found that tobacco was consumed as snuff and chewing tobacco rather than smoking. These behaviours were well entrenched culturally. In addition, farmers consumed tobacco as a hunger suppressant and preferentially planted tobacco crops as it was a high-paying crop, all new perspectives for us in Finland.

On the screening front, the focus was first on establishing cytopathology across the country for accurate diagnosis—training was easy. We still found that referral for treatment of any lesions identified was a challenge, particularly women in rural settings. There were also financial barriers. Back then, extrapolation of screening costs to the whole country would have consumed 5% of the health budget and cancer control would have consumed the whole of the budget assigned for health. Our current focus is improving the performance of services outside of clinical trials through training and establishing robust health information systems. Inequities are a major challenge for the country. There is no mandatory reporting and the ethical dilemma of management of all cancer patients remains in India.

## Knowledge transfer from population research to cancer detection programmes in low- and middle-income countries, Dr Partha Basu

Dr Partha Basu described the role of the International Agency for Research in Cancer (IARC) in supporting cancer research and developing researcher capacity in the three objectives of the agency: describing the occurrence; understanding the causes; evaluate intervention and support implementation. Using the example of early detection of cervical cancer, Basu highlighted IARCs role in establishing visual inspection and treatment of pre-cancerous lesions as a globally recommended intervention by the World Health Organisation (WHO). The feasibility of visual screening of the cervix in developing country settings was demonstrated by IARC and local partners in a multi-site study in India and Africa and published in 2004. A randomised clinical trial in Tamil Nadu, India, illustrated the impact in terms of incidence and mortality in 2007.

Interestingly, these initial outcomes were not replicable at programmatic level. IARC worked alongside implementing teams in a two-step scale up of services across the 32 districts of Tamil Nadu and also in Bangladesh. Implementation research illustrated the outcome gap, but also opportunities for improvement. The South East Asia office of WHO subsequently has worked with regional programme managers to develop a training manual, with facilitators guide, as well as training manuals for programme managers and community health workers. Aiming for consistency of training and on programme management and training of personnel in a holistic manner.

Citing the logistical challenge of routine availability of cryotherapy for treatment of pre-cancers, which was identified as a common barrier to providing a regular service, IARC went on to demonstrate the feasibility of thermal ablation as a treatment alternative using pooled data from Bangladesh, Brazil and India. Manufacturers have also responded, by developing more practical technical solutions, for example a battery-powered thermal ablation tool is now available. WHO will shortly be citing these and other data in a global guideline on thermal ablation for treatment of precancerous lesions of the cervix.

## The Cancer Research Continuum: addressing the increasing burden of cancer by a mission-approach to cancer, Professor U. Ringborg, Sweden

Professor Ringborg, Chairperson of the EurocanPlatform and Director of the Cancer Center Karolinska, Stockholm, picked up earlier conversations on ensuring no country is left behind. He highlighted the increasing challenge faced by all European countries of an aging population, more people living with cancer as a chronic disease and the limited impact of prevention efforts and new research findings in the face of this growing cancer burden. A burden which is having a high economic impact with total healthcare costs in 2009 in Europe now at 126 billion EUR (2009) and direct health cost of cancer amounting to 91.4 billion EUR (2014). Further he illustrated a doubling of cancer drug sales from 9.5 billion to 19.8 billion EUR in Europe between 2005 and 2014.

Responding to the challenge on both fronts, Ringborg presented the capacity of the EurocanPlatform Network of Excellence, which was established in 2011 to structure translational cancer research and led to the creation of Cancer Core Europe in 2014. The critical mass of this network is substantial, annually: 60,000 newly diagnosed cancer patients; 300,000 treated patients; 1,200,000 patient consultations and more than 1500 clinical trials. Coupled with the Cancer Prevention Europe network of ten research agencies, this forms the basis of the Horizon Europe—cancer mission. One of only five missions defined for the region with significant budgetary support. Important says Ringborg is the embracing of innovation, but in the context of quality assured routine cancer care and the long-term outcomes focused goals of the mission.

Importantly, the Horizon mission includes all European countries and adoption of the healthcare system of the Comprehensive Cancer Centre (CCC) approach, with outreach programmes into the community, is instrumental. Cancer Core Europe and Cancer Prevention Europe provide the infrastructure for early translational research and additional consortia of CCCs cover a coherent translational research continuum. Further, a large number of educational programmes in present consortia will be open for young researchers in all EU countries, facilitating exchange of researchers, an Annual Summer School for translational cancer research, educational programmes for the next generation of cancer leaders and bilateral collaboration between Cancer Core Europe centres and centres in central/east EU are other measures to build key research capacities across the region.

## Interactive break out session, Dr. Ulrike Helbig, Germany

The working groups introduced by Dr. Ulrike Helbig, German Cancer Society focused on three key questions:

- Which European learnings can and should be shared beyond Europe?
- Which methods of knowledge transfer for quality improvement are successful?
- How important are these aspects and how much effort should be invested?

## Which European learnings can and should be shared beyond Europe?

All participants agreed that an underlying strategy to develop and improve cancer care should be developed, and best if based on the experience of the participating nationalities, this could be achieved by the implementation of a national cancer control plan (NCCP). Most successful can be followed, when health policies are adjusted to support cancer care.

Each country has to decide how to act specifically depending on the financial, political and structural resources on the one hand and on the other based on the primary needs. It needs to be decided which of the necessary responsibilities can be ensured by which institution, organization or from governmental side.

Clinical care structure and referral mechanisms, as well as research networks including CCCs are needed. Suitable patient-centered models of cancer care should be developed.

Structures to provide accessibility to cancer drugs need to be provided, the WHO listed the essential drugs which should be paid for.

Structural development should be accompanied by the opportunity to use digital data and the dense distribution of mobile phones in some countries. Principles and state of the art criteria (guideline implementation, multi-disciplinarity) with an opportunity to adapt to local capacities and resources should be defined and implemented.

Approaches in education of health professionals as well as of the population can be learned of international institutions (IARC) or transferred bilaterally.

The pressure to serve economic matters differs in countries (health insurance versus no health insurance) and determines quality development in the end. Therefore, a support can already be made by mentioning this to the countries in question.

National Cancer Care Plans (NCCPs) should support the cooperation of public health, clinic engagement (hospital and out-patient services), civil society organizations, and patient groups. In all approaches, cultural and ethical specifications of a country including anti-corruption guidelines should be considered.

In all approaches and aims for development and improvement, the challenge to avoid brain drain by local experts leaving the country and undermining the progress made needs to be avoided.

## Which methods of knowledge transfer for quality improvement are successful?

Strategy plans like National Cancer Care Plans (NCCPs) have proved to be successful. Inevitable of course is the thorough analysis, what is needed and where problems are beforehand.

A practical outline is provided by Cancer Control Planning (European Guide to Cancer Control) and the WHO approach to cancer control.

Core areas of a solid approach to quality in cancer care are guidelines, which should be implemented in cancer centers and, therefore, need specific training to do so and the development of cancer registries (e.g., by IARC) with an adjunct, guided education about their importance for quality control. Cancer centers can be developed by implementation of multi-disciplinarity and quality criteria, here as well, education and development of research in the implementation process and ongoing are crucial.

The way from evidence to decision as well as patient pathways should be adapted to the local circumstances by a local group, and recommendations will be different in low- or middle-income countries. In all processes, timelines need to be shortened.

Therefore, training programmes (e.g., by IARC), personal mentorship with integration of local professionals and institutions (e.g., cancer centers), integration of international activities, research programs, general training, involvement of local authorities should be applied as soon as possible.

Local and collaborative research initiatives support capacity building and staff retention and are a preventive for brain drain.

The role of patient advocacy groups is to demand access, navigate patients and observe outcomes (pan-regional exchange). New technologies facilitate bilateral or multilateral exchange and mentoring. Processes and success can be well supervised in testing pilots and by setting on milestones.

For each activity, sustainability should be pronounced. Once a project has been started, planning has to be made on a long-term basis. Ideally, the responsibility for each project should be transferred to local individuals or organizations to facilitate long-term performance.

## How important are these aspects and how much effort should be invested?

Efforts should be given to each point mentioned above, but experience shows that specifically high effort to secure political support is important. Informing and education of the whole community is the base of quality maintenance of public health.

Another strong focus should be set on the improvement of collaboration and network development, collaborations might be institutionalized. The role of international partnerships is increasingly important.

Networking with centers on patient outcomes is crucial. The integration of research into the processes is a strong driver for capacity building.

Sustainability is in the scope of the current WHO goals. Most oncological affections are chronic diseases with regard to etiology, growth, treatment and course of the disease. Therefore, prevention, screening and early detection as well as diagnosis, acute and long-term treatment and follow-up can only be improved in a long-tem manner.

To secure long-term results, brain drain needs to be avoided.

Rehabilitation, early and late palliative care, cost effectiveness and long-term follow-up are priorities. Analytical processes need to be adjusted according to the local circumstances.

In the end, often it is budget constraints which limit the execution of a detailed NCCP.
