# Correction to: Knowledge transfer as a tool towards improvement of cancer care in low- and middle-income countries. 6th European Roundtable Meeting (ERTM), June 14th, 2019, Berlin, Germany

**DOI:** 10.1007/s00432-021-03709-0

**Published:** 2021-07-02

**Authors:** O. Ortmann, J. Torode, W. Schmiegel, C. Thomssen, M. Hakama, P. Basu, U. Ringborg, U. Helbig, For the participants of the ERTM, Matti Hakama, Partha Basu, Marjetka Jelenc, Josep Borras, Ulrik Ringborg, Julie Torode, Jan-Willem Coebergh, Johannes Bruns, Markus Follmann, Ulrike Helbig, Andreas Hochhaus, Monika Klinkhammer-, Margarete Landenberger, Olaf Ortmann, Wolff Schmiegel, Christian Thomssen

**Affiliations:** 1 German Cancer Society, Berlin, Germany; 2 Department of Gynecology and Obstetrics, University Medical Center Regensburg, Landshuter Straße 65, 93053 Regensburg, Germany; 3 Union for International Cancer Control (UICC), Geneva, Switzerland; 4 University Clinic at the University Hospital Knappschaftskrankenhaus Bochum, Bochum, Germany; 5 University Clinic and Polyclinic for Gynaecology, Martin-Luther University Halle-Wittenberg, Halle (Saale), Germany; 6 The Finnish Cancer Registry, Helsinki, Finland; 7 International Agency for Research On Cancer, World Health Organisation, Lyon, France; 8 EUROCAN Platform, Cancer Center Karolinska, Stockholm, Sweden; 9 Deutsche Krebshilfe, Berlin, Germany

## Correction to: Journal of Cancer Research and Clinical Oncology (2020) 146:1813–1818 10.1007/s00432-020-03209-7

The article “Knowledge transfer as a tool towards improvement of cancer care in low- and middle-income countries. 6th European Roundtable Meeting (ERTM), June 14th, 2019, Berlin, Germany” written by O. Ortmann, J. Torode, W. Schmiegel, C. Thomssen, M. Hakama, P. Basu, U. Ringborg, U. Helbig and for the participants of the ERTM was originally published electronically on the publisher’s internet portal on April 8, 2020 without open access. With the author(s)’ decision to opt for Open Choice the copyright of the article changed to © The Author(s) 2020 and the article is forthwith distributed under a Creative Commons Attribution 4.0 International License, which permits use, sharing, adaptation, distribution and reproduction in any medium or format, as long as you give appropriate credit to the original author(s) and the source, provide a link to the Creative Commons licence, and indicate if changes were made. The images or other third-party material in this article are included in the article’s Creative Commons licence, unless indicated otherwise in a credit line to the material. If material is not included in the article’s Creative Commons licence and your intended use is not permitted by statutory regulation or exceeds the permitted use, you will need to obtain permission directly from the copyright holder. To view a copy of this licence, visit http://creativecommons.org/licenses/by/4.0/. Open Access funding enabled and organized by Projekt DEAL.

